# Joseph Lister (1827-1912): A Pioneer of Antiseptic Surgery

**DOI:** 10.7759/cureus.32777

**Published:** 2022-12-21

**Authors:** Spyros N Michaleas, Konstantinos Laios, Alexandros Charalabopoulos, George Samonis, Marianna Karamanou

**Affiliations:** 1 Department of History of Medicine and Medical Ethics, National and Kapodistrian University of Athens School of Medicine, Athens, GRC; 2 First Department of Surgery, Laiko General Hospital, National and Kapodistrian University of Athens, Athens, GRC; 3 Department of Internal Medicine, University of Crete School of Medicine, Heraklion, GRC

**Keywords:** ­wound healing, microorganisms, spontaneous generation, louis pasteur, sterilization, carbolic acid

## Abstract

Joseph Lister was a prominent British surgeon and medical scientist who established the study of antisepsis. Applying Louis Pasteur’s germ theory of fermentation on wound putrefaction, he promoted the idea of sterilization in surgery using carbolic acid (phenol) as an antiseptic. His method reduced the incidence of wound sepsis and gangrene, which, in turn, reduced the need for amputation. By showing how germs could be prevented from entering the wound, Lister increased the safety of surgical operations and laid the foundations for all subsequent advances in the field.

## Introduction and background

**Figure 1:**
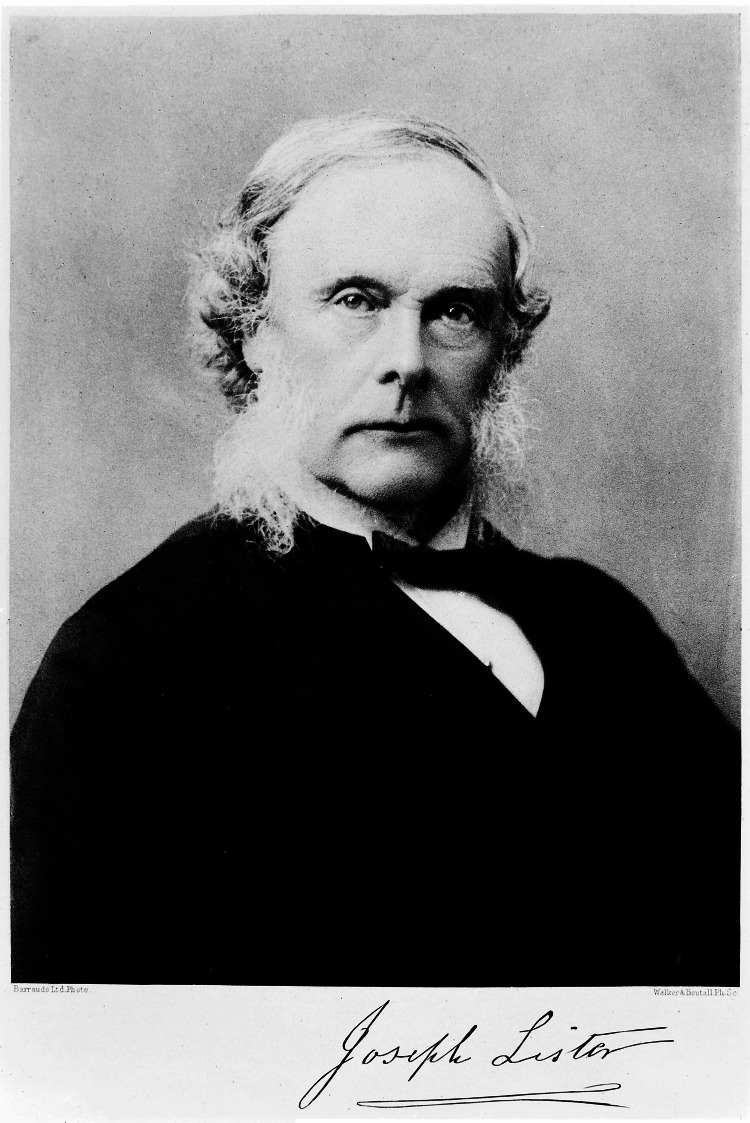
Lister, Joseph, Baron, 1827-1912. Credit: Permission obtained from Wellcome Collection. The collected papers of Joseph, Baron Lister.

The main purpose of this article is to highlight the indisputable contribution of Joseph Lister to modern surgery (Figure 1). Lister was a British surgeon and medical scientist who reformed the art of surgery by introducing the concepts of antiseptics and preventive medicine [1,2]. Interested in wound healing, Lister studied surgical outcomes after sterilizing surgical instruments, the patient’s skin, sutures, and the surgeon’s hands with a chemical substance called carbolic acid (phenol). His experiments were based on research by French chemist and microbiologist Louis Pasteur (1822-1895), who disproved the theory that the spontaneous generation of microorganisms was responsible for diseases [3].

During Lister’s time, surgical knowledge was limited. Microorganisms had been known to be associated with diseases since the 16th century, but no correlation had been found between germs and wound infection. Consequently, bed covers and surgeons’ coats were not washed, and surgical tools were rarely cleaned. In fact, pus formation, or suppuration, was considered part of the natural wound-healing process [4]. Lister made a remarkable observation that wound infection seemed to be associated with exposure to the air. He began successfully applying an antiseptic approach to surgery based on the principles of “no germs, no infection, no disease.” He focused on compound fracture wounds, which at that time often required amputation. His success helped advance the fields of bacteriology, wound treatment, and surgery, reducing mortality caused by postoperative infection and distinguishing Lister as the “father of modern surgery” [4].

## Review

Lister’s life and career

Born in 1827 in Essex, England, Joseph Lister was the fourth child of Joseph Jackson and Isabella Lister. Joseph Jackson was elected Fellow of the Royal Society and helped design and construct the achromatic object lens, which is used in compound microscopes [4-6]. An educated Quaker, Joseph Jackson was a great influence on his son.

Lister’s formal education began in Benjamin Abbott’s Isaac Brown Academy, a private Quaker school in Hertfordshire, and he later attended Grove House School in Tottenham, also a private Quaker school, where he studied language, mathematics, and natural sciences [5]. At age 17, he applied to the University College of London, one of the few institutions in Great Britain that accepted Quakers at that time [7]. As was standard for the time, he first became a wound dresser in January 1851 and then a house surgeon under his professor, John Eric Erichsen (1818-1896), in May 1851 [4,5,8,9]. Lister graduated with a Bachelor of Medicine in 1852 and then obtained a fellowship in the Royal College of Surgeons [4-6,8,10].

After completing his medical education, Lister took the advice of another professor, William Sharpey (1802-1880), and traveled to Edinburgh around 1853 to work under the supervision of eminent surgeon James Syme (1799-1870). In Edinburgh, Lister served as a house surgeon and private assistant to Syme. In 1856, he was elected assistant surgeon to the Royal Infirmary [4-6,8-10]. That same year, Lister married Syme’s eldest daughter, Agnes. Agnes worked as his assistant for the rest of her life, and together they performed several experiments on inflammation and coagulation of blood.

In 1860, Lister was elected a Fellow of the Royal Society and obtained the important position of Regius Professor in Surgery at Glasgow University, where he had a rapidly growing class of students. In 1861, he received privileges at the Glasgow Royal Infirmary [4-6,10], and he continued to receive many honors and prominent awards throughout his career. He passed away on February 10, 1912, at the age of 84, leaving behind a huge legacy in surgical practice [2,4,5].

Pasteur’s influence on Lister

Lister wanted to decrease the rates of wound sepsis that often occurred in hospital settings. He was intrigued by Louis Pasteur’s work, which he learned about from Thomas Anderson (1819-1874), a professor of chemistry in Glasgow. Pasteur had proven that liquids such as milk and juice had much lower rates of fermentation and putrefaction if they were protected from the air, and he concluded that these processes were caused by airborne microbes. This work disproved a popular theory at the time that harmful microorganisms generated spontaneously.

**Figure 2:**
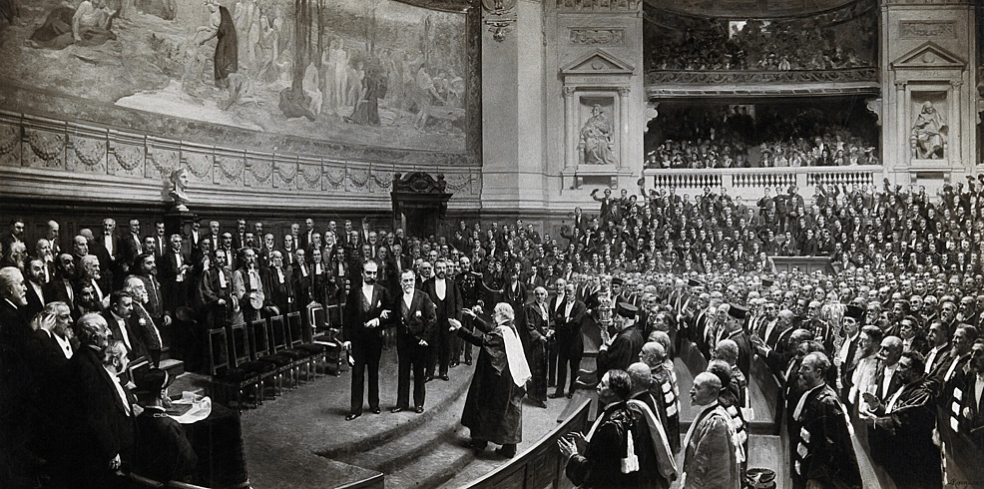
Joseph Lister, Baron Lister acclaims Louis Pasteur at Pasteur’s Jubilee, Paris, 1892. Photograph after a painting by Jean-André Rixens. Credit: Permission obtained from Wellcome Collection.

Lister was greatly influenced by Pasteur. On December 27, 1892, the entire scientific community, including Lister, celebrated Pasteur’s 70th birthday in a crowded amphitheater at the Sorbonne in Paris. University staff, ministers of state, ambassadors, representatives from the Institut de France, and scientists attended. In a picture painted 10 years later by the artist Jean-André Rixens, Lister is ascending the steps to congratulate Pasteur [3,5,6,11-15] (Figure 2).

Influenced by Pasteur’s work, Lister hypothesized that microorganisms in the air caused wound infection and suppuration. At the time, because there was no differentiation between pathogenic microorganisms and harmless ones, Lister was strongly convinced that the air in medical settings should be disinfected. In the postsurgical setting, Lister argued, infections could be prevented by placing an antiseptic shield between the wound and the air surrounding it. It was even speculated that every disease might be prevented by removing or killing airborne germs, such as by filtration, heat, or chemicals [3,4,6,11-15].

Lister’s antiseptic method and spray apparatus

As filtration and exposure to heat were unsuitable for treating human skin, Lister suggested a chemical substance [3,5,13-17]. In 1865, carbolic acid, commonly known as creosote, was used to disinfect compound fractures. Lister experimented with this substance by dipping a pad in carbolic acid solution and then applying it on the wound of an 11-year-old boy. Four days later, when replacing the pad, he saw that no infection had appeared and the boy’s bones had begun to fuse with no sign of suppuration.

From 1865 to 1867, Lister treated 11 more cases of compound fractures, nine of which remained free of infection, one of which needed amputation, and one in which the patient died due to secondary hemorrhage. The results of those experiments were published in six articles in The Lancet from March 1867 to July 1867 [4-6,11,16-18]. In 1867, Lister adjusted his method, applying carbolic acid as a lotion directly to the raw wound in surgery. He also applied an antiseptic paste of carbolic acid to the sutured wound, with excellent results, which he shared with the British Medical Association in Dublin that same year [6]. Based on his experimental data, Lister advised surgeons to wear clean gloves and wash their hands and instruments before and after procedures using a 5% carbolic acid solution. He also suggested not using porous materials for the handles of medical instruments [5,6,9,11,16,17].

**Figure 3:**
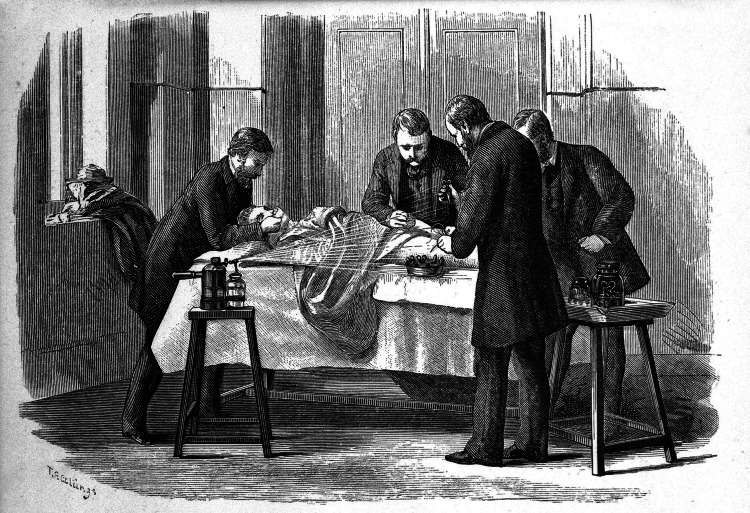
The antiseptic system in practice in an operating room. In: Antiseptic surgery. Its principles, practice, history, and results by William Watson Cheyne (1882). Credit: Permission obtained from Wellcome Collection.

From 1871 to 1887, Lister used a 1:100 dilution of carbolic acid lotion to spray the operating room, believing that the vapor and droplets would be powerful enough to extinguish all germs [5,6,11,12] (Figure 3).

**Figure 4:**
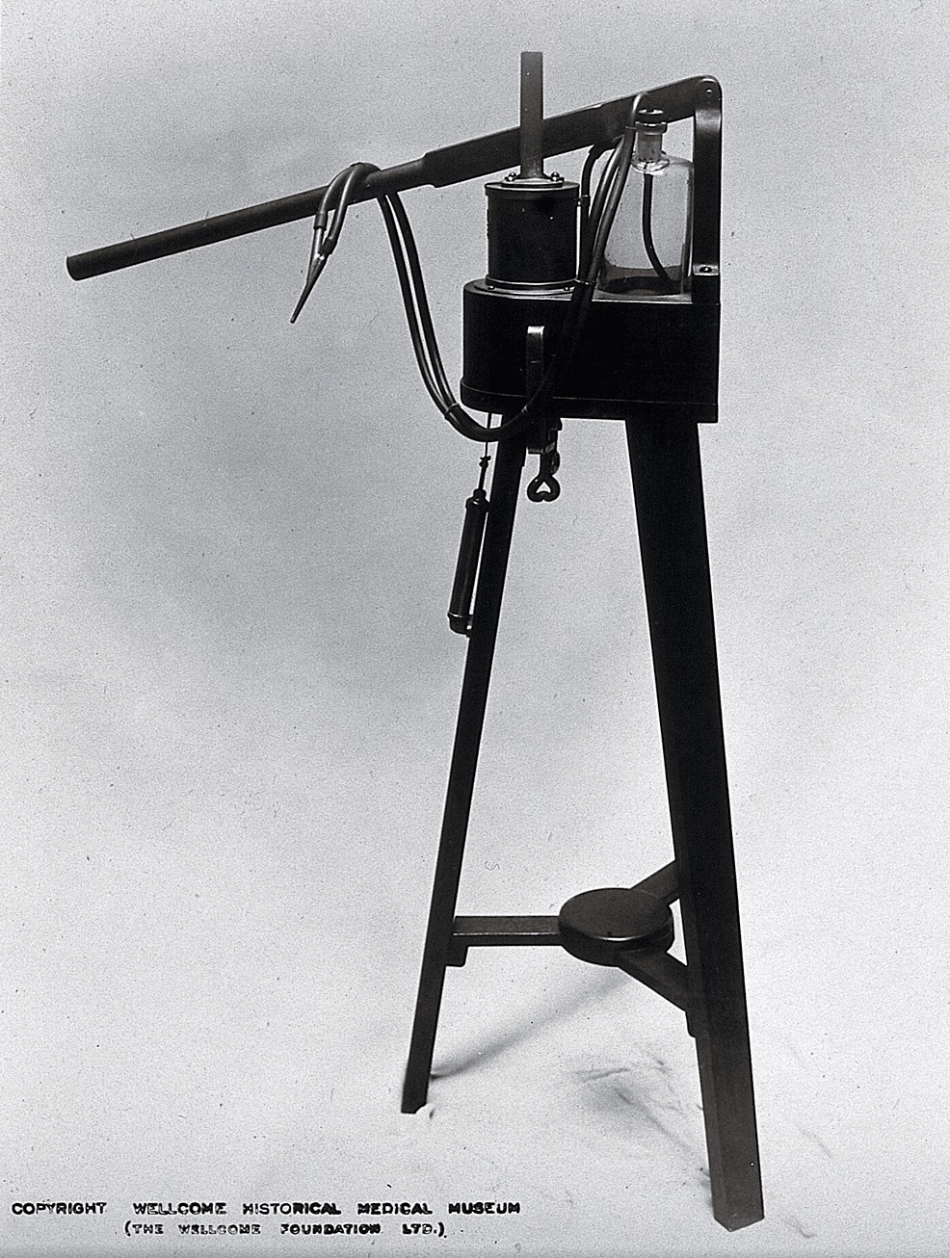
Donkey engine used by Joseph Lister. Photograph, 1927. Credit: Permission obtained from Wellcome Collection.

The spray apparatus itself went through several iterations. At first, a small handheld device was used, but it required the help of an assistant, so a foot spray was later introduced. Both the hand and foot spray devices were cumbersome to operate, so the next model was placed on a tripod and operated using a long handle (Figure 4).

**Figure 5:**
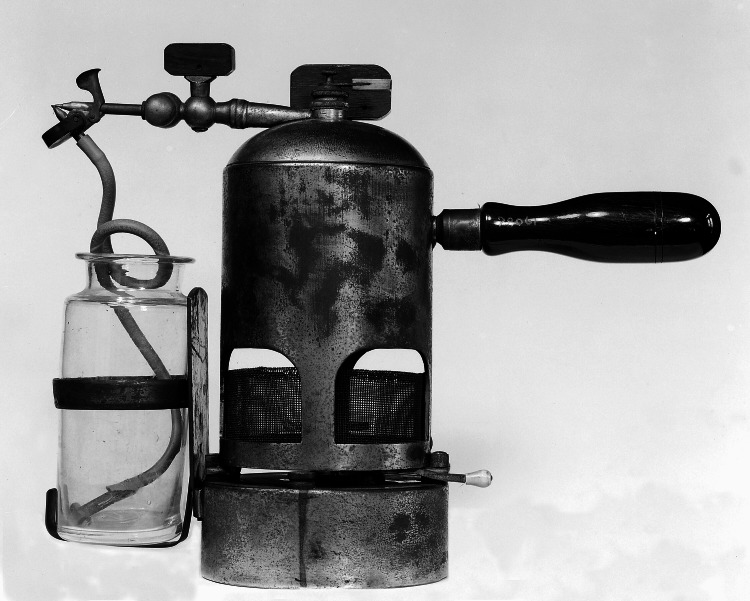
The Lister carbolic spray. Credit: Permission obtained from Wellcome Collection.

That model, nicknamed the “donkey engine,” was replaced by a steam spray device [5,6] (Figure 5).

Despite its advantages, carbolic acid was also known to cause damage to living tissues. For this reason, Lister began to gradually reduce its strength. It is, nevertheless, likely that many of his patients suffered carboluria, carbolic acid poisoning [5,11]. Even with this controversial side effect, the carbolic acid spray was used by scientists and surgeons all over the world. Those who worked with Lister or had observed his methods firsthand attested to the efficacy of his antiseptic method. However, the method was not accepted by those who tried to use it negligently [5,6].

## Conclusions

Joseph Lister helped introduce germ theory and laid the foundation for the use of antiseptics in the practice of medicine and surgery. Today, asepsis and sterile techniques have replaced antisepsis as the principal method in combating wound infection. Lister’s observations and recommendations helped revolutionize surgical practice, making surgery and wound healing safer for patients.
